# Clozapine counteracts a ketamine-induced depression of hippocampal-prefrontal neuroplasticity and alters signaling pathway phosphorylation

**DOI:** 10.1371/journal.pone.0177036

**Published:** 2017-05-04

**Authors:** Marion Rame, Dorian Caudal, Esther Schenker, Per Svenningsson, Michael Spedding, Thérèse M. Jay, Bill P. Godsil

**Affiliations:** 1 Laboratoire de Physiopathologie des Maladies Psychiatriques, UMR_S894 Inserm, Centre de Psychiatrie et Neurosciences, Paris, France; 2 Université Paris Descartes, Sorbonne Paris Cité, Paris, France; 3 Department of Clinical Neuroscience, Center for Molecular Medicine, Karolinska Institute, Stockholm, Sweden; 4 Institut de Recherches Servier, Croissy-sur-Seine, France; 5 Spedding Research Solutions SAS, Le Vesinet, France; Technion Israel Institute of Technology, ISRAEL

## Abstract

Single sub-anesthetic doses of ketamine can exacerbate the symptoms of patients diagnosed with schizophrenia, yet similar ketamine treatments rapidly reduce depressive symptoms in major depression. Acute doses of the atypical antipsychotic drug clozapine have also been shown to counteract ketamine-induced psychotic effects. In the interest of understanding whether these drug effects could be modeled with alterations in neuroplasticity, we examined the impact of acutely-administered ketamine and clozapine on *in vivo* long-term potentiation (LTP) in the rat’s hippocampus-to-prefrontal cortex (H-PFC) pathway. We found that a low dose of ketamine depressed H-PFC LTP, whereas animals that were co-administrated the two drugs displayed LTP that was similar to a saline-treated control. To address which signaling molecules might mediate such effects, we also examined phosphorylation and total protein levels of GSK3β, GluA1, TrkB, ERK, and mTOR in prefrontal and hippocampal sub-regions. Among the statistically significant effects that were detected (a) both ketamine and clozapine increased the phosphorylation of Ser9-GSK3β throughout the prefrontal cortex and of Ser2481-mTOR in the dorsal hippocampus (DH), (b) clozapine increased the phosphorylation of Ser831-GluA1 throughout the prefrontal cortex and of Ser845-GluA1 in the ventral hippocampus, (c) ketamine treatment increased the phosphorylation of Thr202/Tyr204-ERK in the medial PFC (mPFC), and (d) clozapine treatment was associated with decreases in the phosphorylation of Tyr705-TrkB in the DH and of Try816-TrkB in the mPFC. Further analyses involving phosphorylation effect sizes also suggested Ser831-GluA1 in the PFC displayed the highest degree of clozapine-responsivity relative to ketamine. These results provide evidence for how ketamine and clozapine treatments affect neuroplasticity and signaling pathways in the stress-sensitive H-PFC network. They also demonstrate the potential relevance of H-PFC pathway neuroplasticity for modeling ketamine-clozapine interactions in regards to psychosis.

## Introduction

The relative failure in developing new therapeutic drugs for psychiatric disorders that act upon single brain targets has encouraged research that examines the full range of variation—from normal to abnormal—in brain circuits implicated in the pathophysiology of these psychiatric disorders [1]. Partly because stress is a precipitating factor for psychiatric symptoms [2–4], and partly because it strongly impacts neuroplasticity in specific brain circuits [5], understanding the relationship between therapeutics and stress-sensitive neuroplasticity has been a vigorously-investigated research domain. The hippocampus-to-PFC pathway (H-PFC) consists of neurons originating in the ventral CA1/subicular regions that project to the prefrontal cortex [6, 7]. We have previously demonstrated that the H-PFC has impaired neuroplasticity after exposure to acute or chronic stressors [8, 9], and we have identified antidepressant and antipsychotic drugs that restore this neuroplasticity in stress-exposed animals [8, 10]. More recently, there has been a growing understanding for how the H-PFC contributes to cognitive function and emotional regulation [11–16], and we have argued that pathophysiology in the H-PFC is relevant to multiple psychiatric disorders, including schizophrenia and major depression [17], although, undoubtedly, other connected brain regions and pathways are also engaged [18–21].

In recent years, there has been expanding interest in the brain mechanisms related to ketamine because a low-dose treatment of the drug has been shown to reduce depressive symptoms in treatment-resistant patients with major depression [22, 23]. It has also been demonstrated that neural activity in the H-PFC pathway is necessary for the anti-depressant-like effect of ketamine in an animal model of depression [13]. Interestingly, ketamine has also been investigated in relation to models of schizophrenia because a similar single low dose of the drug exacerbates schizophrenic symptoms in patients [24] and induces schizophrenic-like symptoms in healthy humans [25], such as cognitive impairments resembling dissociative thought disorder [26]. Thus, a sub-anesthetic dose of ketamine appears to have both early and delayed effects on psychiatric symptoms, in that the dissociative or schizophrenic-like effects of ketamine typically peak 30–40 minutes after drug treatment [24, 27], while the antidepressant effects continue for typically several days after the drug has been metabolized [23]. The atypical anti-psychotic drug clozapine (which is often used to treat the psychotic symptoms of treatment-resistant schizophrenic patients [28]) has also been shown to reverse ketamine-induced psychotic symptoms in healthy humans [29], and to reduce the ketamine-induced exacerbation of positive symptoms in patients [30]. The mechanisms for these effects are not well understood, but evidence from animals studies have shown that clozapine counteracts several ketamine-induced phenomena, including: alterations in medial PFC (mPFC) glutamate metabolism [31] and oxygenation levels [32], deficits in sensory-evoked gamma oscillations [33], disruptions in paired-pulse inhibition [33, 34], and in the release of serotonin in the PFC [35].

Taking into account these findings, as well as the hypothesis that alterations in H-PFC plasticity are relevant for modeling pathophysiology, we became interested in the interrelation of ketamine and clozapine on H-PFC pathway function. Indeed, we previously observed previous that a low-dose of clozapine (0.3 mg/kg) was optimal for modifying frontal cortex theta rhythms [36, 37], which are considered important for long-range connectivity between the hippocampus and mPFC [38, 39], and a post-stress treatment of clozapine at this dose also protected H-PFC pathway plasticity from stress-induced disruption [10]. Thus, considering that the early impact of ketamine and clozapine on neuroplasticity may be germane to understanding their pro- and anti-psychotic actions, as well as to understanding ketamine’s longer-lasting antidepressant effects, we examined the influence of ketamine and clozapine on *in vivo* long-term potentiation (LTP) in the rat H-PFC pathway that was induced during the interval in which ketamine produces its schizophrenic-like effects. There has also been controversy regarding the effect of ketamine treatments on brain signaling pathways [40], and there has been a dearth of information concerning how a low-dose of clozapine influences these cascades. Moreover, there is sparse data addressing how clozapine may counter ketamine effects within these signaling cascades. So, to further the understanding of how ketamine and clozapine drug treatments alter signaling cascades within the H-PFC network, we measured the phosphorylation and total protein levels of GSK3β, GluA1, TrkB, ERK, and mTOR in the mPFC and lateral PFC (latPFC), as well as the dorsal and ventral hippocampus (DH and VH).

## Materials and methods

### Animals

Experimentally-naïve adult male Sprague-Dawley rats (300–400g; Charles River, France), maintained in a temperature-controlled facility (22 ± 1°C; 12/12h light/dark schedule), were used. Animals were housed in pairs, they had free access to food and water, and were maintained at least one week after delivery from the supplier before use. Procedures were performed during the light cycle, and they were conducted in conformity with the EU Directive 2010/63/EU for animal experiments. The institutional ethics committee approved all the experimental procedures (CEEA Paris Descartes Comité 34) under protocol number 01067.02. All efforts were made to minimize animal suffering and to reduce the number of animals used.

### Drugs

Except for the intravenous ketamine experiment (S1 Fig), all drugs were administered with intraperitoneal (ip) injections. Sodium pentobarbital (Ceva Sante Animale, France) was administered at anesthetic doses for all animals. The first injection was 60 mg/kg, and thereafter, animals in the electrophysiology experiments received additional supplements as necessary. Ketamine hydrochloride powder (LGC Standard, France; (±)-2-(o-Chlorophenyl)-2-(methylamino) cyclohexanone hydrochloride) and clozapine powder (Novartis; 8-chloro-11-(4-methylpiperazin-1-yl)-5H-dibenzo[b,e][1,4]diazepine) were dissolved in 0.9% NaCl.

### Electrophysiological experiments

Rats were deeply anesthetized with sodium pentobarbital and placed in a stereotaxic frame while body temperature was maintained at 37°C with a homeothermic warming blanket. Using stereotaxic surgical procedures, burr holes were drilled into the skull above the mPFC and VH. A single recording electrode (64-μm diameter, two nickel-chrome wires) was positioned in the prelimbic cortex (3.3 mm anterior to bregma, 0.8 lateral to the midline) and a bipolar concentric stainless steel stimulating electrode (150-μm outer diameter with a 300-μm tip separation) was lowered into the ipsilateral CA1 region of the VH (posterior to bregma; 5.5 lateral to the midline). Electrical stimulation of the ventral CA1/subicular region evoked a characteristic monosynaptic, negative-going, excitatory postsynaptic potential (PSP) in the prefrontal cortex with a peak latency of 18–22 ms [41]. The final dorsal/ventral positions of the stimulating and recording electrodes were selected by locating the combination of dorsal-ventral positions that yielded the largest field potential amplitude. As such, the final dorsal-ventral positons for the tips of the stimulating and recording electrodes (which were held constant thereafter) were within the ranges of 4.5–6.0 and 3.0–3.8 mm below the cortical surface, respectively. Test pulses (100 ms) were delivered every 30 s at an intensity that evoked 70% of the maximum response (range: 300–500 mA). At this intensity, the field potential most likely reflects summated PSPs. High-frequency stimulation (HFS) to induce LTP was delivered at the test pulse intensity and consisted of two series of 10 trains (250 Hz, 200 ms, at 0.1 Hz) that were spaced 6 min apart. Postsynaptic potential amplitudes were analyzed using A/Dvance software (McKeller Designs, Canada), expressed as a percentage change of the mean response over a 30 min baseline period and presented in figures as the mean ± SEM for 2-min epochs. Drug were administered 40 minutes prior to the delivery of HFS (10 minutes before the start of baseline recordings). For the pilot experiment involving intravenous drug administration, a catheter was surgically implanted that penetrated the femoral artery, and ketamine was perfused through the catheter with a glass syringe.

### Western blotting measurements

Separate groups of rats were used to examine the influence of ketamine and/or clozapine treatments on proteins sampled from the mPFC, latPFC, DH and VH. Rats received a sequence of three ip injections. The first and second injections were some combination of saline, ketamine, or clozapine that varied by group. The control group received two injections of saline. The ketamine group received an injection of ketamine followed by an injection of saline. The clozapine group received an injection of saline followed by an injection of clozapine. The ketamine + clozapine group received an injection of ketamine followed by an injection of clozapine. The third injection was sodium pentobarbital, which was administered in order to retain continuity with the electrophysiological experiments, as well as with previous reports [8, 42], including studies that measured ex vivo protein phosphorylation events [43, 44]. After the injections, rat body temperature was maintained with homoeothermic warming blankets (37°C) until the animal was killed by decapitation 30 min later, whereafter its whole head was snap frozen in liquid nitrogen as previously described [45]. Brain tissue was removed from the skull and the frontal cortices were separated into medial and lateral parts, and the dorsal and ventral hippocampus were also separated manually. All samples were frozen on dry ice and stored at -80°C until processed.

Frozen tissue samples were sonicated in 1% sodium dodecyl sulfate (SDS), 10 mM NaF, transferred to Eppendorf tubes and boiled for 10 min. The protein concentration in each sample was determined with a BCA-based kit (Pierce, Rockford, IL, USA). Equal amounts of protein (20 μg) of each sample were loaded onto 9% or 15% acrylamide gels. The proteins were separated by SDS-PAGE then transferred to Immobilon-P Polyvinylidene Difluoride membrane (Sigma) on ice for 3 hours at 400mA, 100V. The membranes were incubated for 1 h at room temperature with 5% (w/v) dry milk in TBS-Tween 20 then for 2 h with primary antibodies. Immunoblotting was carried out using different phosphorylation-state-specific antibodies: P-Ser831-GluA1 (1:500, Millipore, AB5847), P-Ser845-GluA1 (1:500, Upstate, 06–773), P-Thr202/Tyr204-p44/42-ERK (1:1000, Cell Signaling, 9101L), P-Ser9-GSK3β (1:1000, Cell Signaling, 9323S), P-Tyr705-TrkB (1:1000, Abcam, ab52191), P-Ser2481-mTOR (1:1000, Cell Signaling, 2974S), and P-Tyr816-TrkB (1:1000, Abcam, ab75173). Antibodies which were not phosphorylation state-specific were used to estimate total levels of GluA1 (1:1000, Upstate, 06–306), p44/42-ERK (1:1000, Cell Signaling, 9107S), GSK3β (1:1000, Cell Signaling, 9832), mTOR (1:1000, Cell Signaling, 2972S), and TrkB (1:1000, Millipore, 07–225). Membranes were washed three times with 0.1% TBS-Tween 20 and incubated with secondary HRP anti-rabbit antibody or HRP anti-mouse antibody (dilutions 1:1000, Dako Sweden AB, Stockholm, Sweden) for 1 h at room temperature. At the end of the incubation, membranes were washed three times with TBS-Tween 20 and the immunoreactive bands were detected by chemiluminescence (Clarity Western ECL kit, Bio-Rad Laboratories, Inc., Berkeley, CA, USA). A series of primary, secondary antibody dilutions and exposure times were used to optimize the experimental conditions for the linear sensitivity range of the autoradiography films (Kodak Biomax MR). Films were scanned and the density of each band was quantified using the NIH ImageJ 1.29 software. The ratio between phosphorylated form levels and total protein levels.

### Statistical analyses

Data from the physiology experiments were entered into either a t-test, one-way ANOVA, or a repeated-measured ANOVAs, and significant F-tests were followed up with planned comparisons where appropriate. To estimate phosphorylation levels, statistical analyses were performed with the within-subject ratio data (phospho-protein measurement/total protein measurement). Owing to concerns regarding between-group heterogeneity of variance, except where noted, the ratio data from each residue (split by sub-region) were entered into separate one-way permutation tests (“approximative general independence test” of the R statistical programing language, coin package, v1.1.3). Significant overall tests were followed up with permutation-based planned comparisons (control vs ketamine, control vs clozapine, and ketamine vs ketamine + clozapine), and to control for the false discovery rate, p-values were adjusted using the Benjamini—Hochberg procedure [46]. For all analyses the criterion for significance was p < 0.05. p-values ≥ 0.05 but < 0.10 were referred to as non-significant trends. Total protein data were also analyzed separately, using the same method. For the relative effect size analysis of the phosphorylation/total protein ratio data, two versions of the *Cohen’s d* effect size were used: one that employed the pooled standard deviation of the control and experimental group, and another that used only the standard deviation of the control group. Calculations and statistical tests were computed with a combination of Statistica software (v12) and customized scripts written with R (v3.3.2) statistical programming language software.

## Results

### Electrophysiology

The results from a pair of pilot experiments conducted in our laboratory suggested that (a) pre-treatment with 0.3 mg/kg clozapine did not markedly alter the induction or expression of LTP in the H-PFC pathway (S1A and S1B Fig), whereas (b) pre-treatment with 10 mg/kg ketamine was associated with a depression of this form of neuroplasticity (S1C and S1D Fig). We then followed up these pilot experiments with one that was designed to address two issues: (1) to confirm whether a low, non-anesthetic dose of ketamine depresses the induction of activity-dependent synaptic plasticity in the H-PFC pathway, and (2) to determine whether a low dose of clozapine might prevent the hypothesized influence of ketamine on the LTP. This was accomplished by examining the physiological responses of three groups of rats that were treated with either saline (n = 8), 10 mg/kg ketamine (n = 8), or 10 mg/kg ketamine + 0.3 mg/kg clozapine (n = 8) 40 min prior to the induction of HFS.

**Fig 1:**
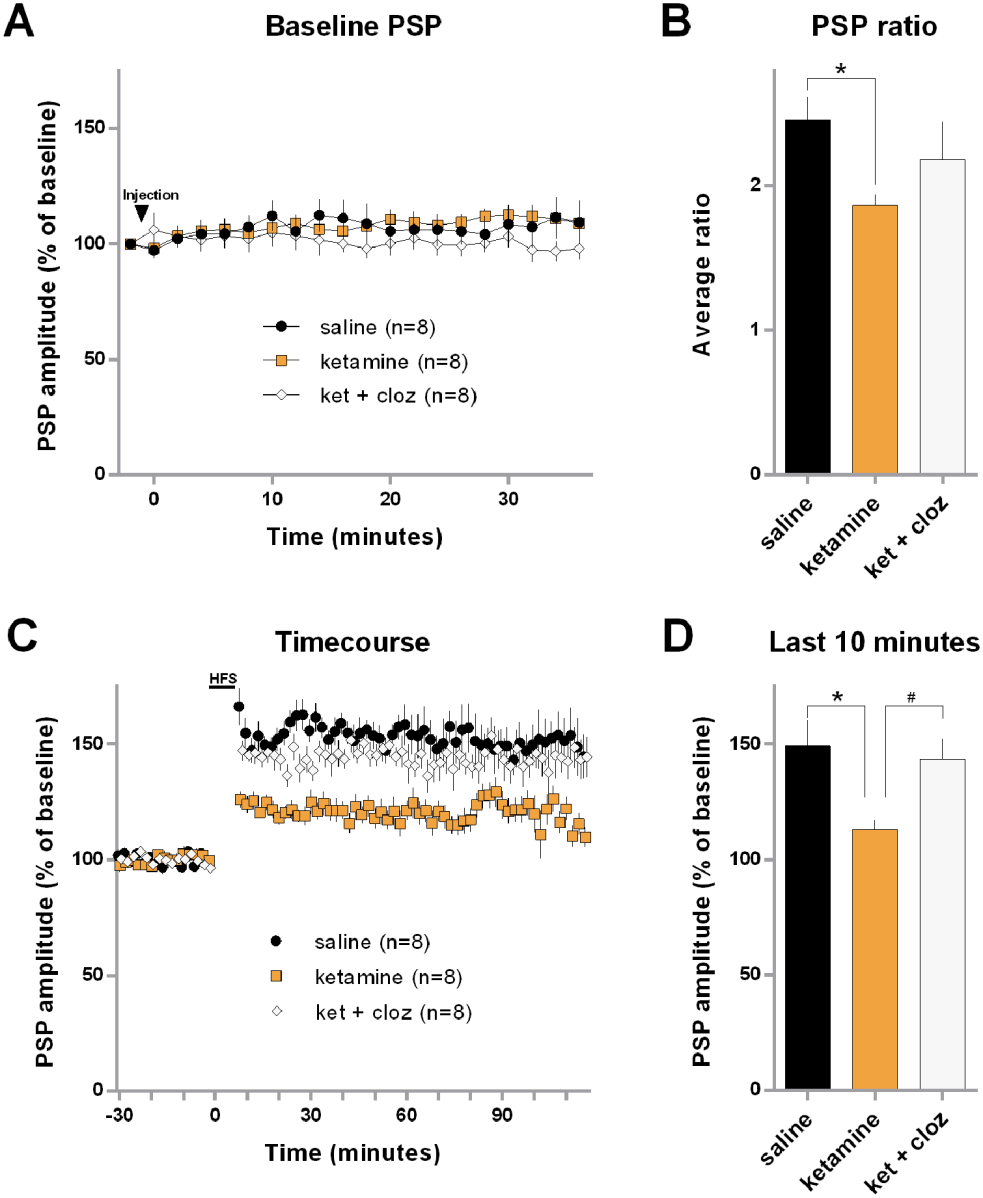
Clozapine protected H-PFC synaptic plasticity that was depressed by acute ketamine. Data recorded in the mPFC from groups of rats administered saline, ketamine (10 mg/kg, ip), or ketamine and clozapine (0.3mg/kg, ip) 40 minutes prior to the induction of LTP via high-frequency electrical stimulation (HFS) delivered to the ventral hippocampus/subicular region. (A) Average post-synaptic potentials (PSP) of each group during the interval from two min before the injections until the end of the baseline period. Data are shown in 2 min bins and were normalized with respect to the PSP of each group that preceded the injection. (B) Average ratio of the PSPs measured after and before the first series of trains of the HFS protocol (first post-train PSP/last pre-train-PSP). (C) Timecourse data for each group showing average PSP in 2 min bins before and after HFS. For each group, data were normalized with respect to the 30-min baseline interval. (D) Mean PSP of each group during the last 10 minutes of the post-HFS interval. For each group, data were normalized with respect to the 30-min baseline interval. Error bars represent standard error of the means. * indicates a statistically significant group difference compared to the saline control. # indicates a significant group difference between the ketamine-alone and ketamine + clozapine groups.

To examine the influence of the ketamine and ketamine + clozapine treatments on baseline PSP amplitudes, evoked PSPs recorded during the period spanning from just before the injections until just before the induction of HFS were analyzed. As shown in Fig 1A, on average, both the saline control and ketamine-alone groups displayed a gradual increase in PSP amplitude across the interval (control = 9.4% ± 18; ketamine = 8.8% ± 10). The ketamine + clozapine group displayed a slight average decrease in PSP amplitude over the interval (-2.1% ± 10). These PSP data were entered into a repeated-measure ANOVA with a between-subject factor of Group and a within-subject variable of Bin (19 2-minute bins). This test did not detect any statistically significant effects (Group: F_2,21_ < 1; Bin: F_18,378_ < 1; Group × Bin interaction: F_36,378_ = 1.24, p = 0.167), which is consistent with the idea that the three groups displayed similar baseline responses to the drug treatments prior to the induction of LTP.

To estimate the immediate effect of the ketamine and ketamine + clozapine treatments on H-PFC PSP potentiation, a ratio was calculated using PSP data from each rat from just after and just before the first series of trains of HFS [(amplitude of PSP recorded immediately after the first series of trains of HFS)/(amplitude of the last PSP of the baseline interval)]. As shown in Fig 1B, the rats in the control group displayed the largest, while the ketamine-alone group displayed the smallest average ratio of the three groups, respectively. These data were entered into a one-way ANOVA, which indicated only a non-significant trend for group differences (Group: F_2,21_ = 2.62, p = 0.096). However, by using a post-hoc comparison in the form of a t-test with a Bonferroni-adjusted p-value, a statistically significant difference was detected between the control and ketamine-alone groups (t(14) = 3.32, p = 0.010). These results suggest that the ketamine-alone treatment decreased the magnitude of early PSP potentiation occurring after the first series of HFS trains.

Fig 1C shows the timecourse of PSP data from before and after HFS, with the data normalized to the level of the 30-min baseline interval. The HFS protocol produced a lasting increase of PSP amplitudes in all three groups. The ketamine-alone treated animals had the least robust increase, however, whereas the ketamine + clozapine group exhibited LTP levels more similar to the saline group. The post-HFS data were submitted to a repeated-measures ANOVA with a between-subject factor of Group and a within-subject variable of Block. This analysis revealed a statistically significant effect of Group (F_2,21_ = 12.53, p = 0.00026), but not for Block (F-value < 1), and the Group × Block interaction was also not significant (F-value < 1). Planned comparisons confirmed that averaging across the interval, the ketamine group was decreased compared to both the saline and ketamine + clozapine groups. Additionally, a one-way ANOVA of the average data from the last 10 minutes of the post-HFS interval indicated a significant effect (F_2,21_ = 5.24, p = 0.014), and planned comparisons confirmed that the ketamine group exhibited smaller amplitude PSPs compared to both the saline and the ketamine + clozapine groups at the end of the sampling interval (Fig 1D). These results indicate that a 10 mg/kg ketamine dose depressed LTP in the H-PFC pathway. Furthermore, a low dose of clozapine was sufficient to prevent the ketamine-induced depression of H-PFC LTP.

Notably, we did not observe similar ketamine-induced effects in groups of animals treated with either 5 mg/kg or 25 mg/kg doses of the drug (S2 Fig), which indicates the acute ketamine effect was dose dependent. We had also observed a similar dose-dependent pattern in rats that received chronic ketamine treatments prior to electrophysiological testing (S2 Fig). Together, these findings support the idea that 10 mg/kg ketamine may be the effective dose for modulating H-PFC pathway plasticity. Phencyclidine (another “open channel” non-competitive NMDA blocker) did not appear to have a U-shaped dose dependency curve with respect to the compound’s inhibition of H-PFC LTP (S3 Fig).

### Western blotting measurements

#### Protein phosphorylation

While the impact of ketamine on prefrontal and hippocampal signaling has received much attention, there have been discrepant results in the literature [40], and the role of various pathways in ketamine-related signaling remains debatable. There is also less information concerning how a low dose of clozapine influences signaling in these regions, just as the mechanisms by which clozapine might counteract ketamine-induced effects is not well understood. Our electrophysiological findings raised intriguing questions about the relationship between ketamine and clozapine on H-PFC neuroplasticity. Therefore, to inform the understanding for how these drugs influence signaling pathways in prefrontal and hippocampal regions, we performed phosphorylation and total protein measurements on tissue collected from the mPFC, latPFC, DH, and VH of experimentally-naïve rats treated with saline, ketamine, clozapine, or both ketamine and clozapine. The proteins GSK3β, TrkB, ERK, and mTOR were included in the analysis because they have all been linked to the TrkB pathway, which has been implicated in ketamine-related signaling (e.g [47, 48]). The GluA1 measurements were included because our previous findings had implicated this receptor subunit in the prevention of stress-induced disruptions of H-PFC LTP [8, 43, 49]. The specific phosphorylation sites were Ser9-GSK3β, Ser831-GluA1, Ser845-GluA1, Tyr705-TrkB, Tyr816-TrkB, Thr202/Tyr204-ERK, and Ser2481-mTOR. The first steps of our analysis focused on the hypothesis that the mechanism of ketamine-clozapine interaction might be related to opposing effects of these two drugs at a single phosphorylation site. Therefore, to estimate each individual drug effect, the groups treated with either ketamine-alone or clozapine-alone were contrasted with the saline-treated control. We also tested for clozapine-reversal effects by contrasting the ketamine + clozapine group with the ketamine-alone group. Images of the immunoblots from these measurements can be viewed in S4 Fig.

**Fig 2:**
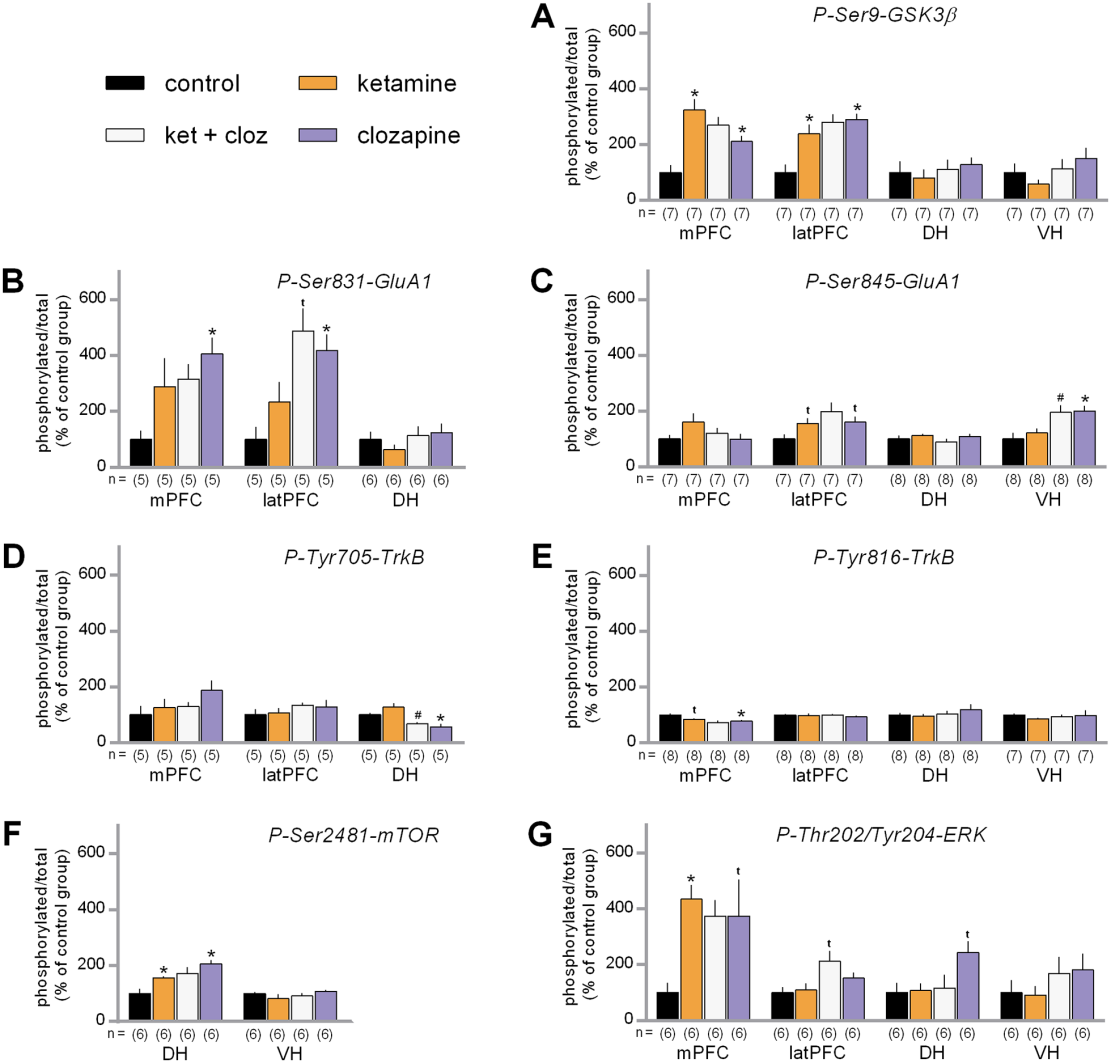
Influence of ketamine and clozapine on the phosphorylation of PFC and hippocampal signaling proteins. Measurements were taken from tissue collected from the medial prefrontal cortex (mPFC), lateral prefrontal cortex (latPFC), dorsal hippocampus (DH) and ventral hippocampus (VH) 30 minutes after the drug treatments. (A) Average phosphorylation levels of Ser9-GSK3β. (B) Average phosphorylation levels of Ser831-GluA1. (C) Average phosphorylation levels of Ser845-GluA1. (D) Average phosphorylation levels of Tyr705-TrkB. (E) Average phosphorylation levels of Tyr816-TrkB. (F) Average phosphorylation levels of Ser2481-mTOR. (G) Average phosphorylation levels of Thr202/Tyr204-ERK. Data are shown normalized with respect to the saline control group. The sample sizes of each group are shown underneath the x-axis. Error bars represent standard error of the means. * indicates a significant group difference compared to the saline control. # indicates a significant group difference compared to the ketamine-only group. t indicates a non-significant trend.

Visual inspection of the data (Fig 2A) suggested both ketamine and clozapine increased Ser9-GSK3β phosphorylation in each prefrontal region, whereas in the hippocampus ketamine may have decreased its phosphorylation. The statistical analysis confirmed significant overall effects in the mPFC (maxT = 3.40, p = 0.0008) and latPFC (maxT = 3.73, p = 0.0003), with significant planned comparisons for each drug in both prefrontal regions (adjusted p-values < 0.017). No differences were detected between the ketamine and ketamine + clozapine groups in the PFC, however (adjusted p-values > 0.29). In contrast, there were no statistically significant effects in the DH (maxT = 0.86, p = 0.80) or VH (maxT = 1.65, p = 0.31). These results support the idea that both ketamine and clozapine caused robust increases of Ser9-GSK3β phosphorylation in the PFC, but not the hippocampus.

Examination of the data shown in Fig 2B suggested both ketamine and clozapine increased Ser831-GluA1 phosphorylation in each prefrontal region, whereas in the DH ketamine may have slightly decreased its phosphorylation. VH data were not included in the analysis because only 3 cases per group were collected. The analysis confirmed significant overall effects in both the mPFC (maxT = 2.58, p = 0.025) and latPFC (maxT = 2.62, p = 0.016), with statistically significant planned comparisons for clozapine in each region (adjusted p-values < 0.023), but not for ketamine (adjusted p-values > 0.14). No significant differences were detected between the ketamine and ketamine + clozapine groups in the PFC (adjusted p-values: mPFC = 0.84; latPFC = 0.082). Unlike the prefrontal regions, there was no statistically significant effect in the DH (maxT = 1.48, p = 0.41). These results indicate that clozapine increased Ser831-GluA1 phosphorylation in the prefrontal cortex. There was also some indication for the possibility that ketamine did the same, but those groups displayed a relatively high degree of within-group variability.

As seen in Fig 2C, ketamine slightly increased Ser845-GluA1 phosphorylation in PFC, while clozapine appeared to increase phosphorylation for this residue in the latPFC and VH. The statistical analysis indicated that there was no statistically significant effect in the mPFC (maxT = 2.12, p = 0.11), and a marginally significant overall effect in the latPFC (maxT = 2.45, p = 0.040). The planned comparisons revealed only non-significant trends for increased phosphorylation in both the ketamine and clozapine-alone groups in the latPFC, however (adjusted p-values = 0.07). In the DH there were also no statistically significant effects detected (maxT = 1.54, p = 0.37). In contrast, in the VH there was a marginal statistically significant overall effect (maxT = 2.43, p = 0.046), along with a significant planned comparison for increased clozapine-induced phosphorylation (adjusted p = 0.020). There was also a marginally significant planned comparison indicating that the ketamine + clozapine group displayed increased phosphorylation compared to the ketamine-alone control (adjusted p = 0.045). Together, these results indicate clozapine increased Ser845-GluA1 phosphorylation in the VH.

The data for Tyr705-TrkB are shown in Fig 2D. Visual inspection suggested that clozapine treatment may have increased phosphorylation in the mPFC, but decreased it in the DH. VH data were not included in the analysis because only 4 cases per group were collected. The statistical analysis indicated no statistically significant overall effects in the mPFC (maxT = 1.98, p = 0.16) or latPFC (maxT = 1.04, p = 0.73), however. In the DH, a statistically significant overall effect was detected (maxT = 2.86, p = 0.0051). Planned comparisons confirmed that clozapine treatment decreased phosphorylation in the DH compared to the saline control, and ketamine + clozapine had decreased phosphorylation compared to the ketamine-alone group (adjusted p-values = 0.033). These results show that clozapine treatment decreased Tyr705-TrkB phosphorylation in the DH.

With regards to Tyr816-TrkB, visual inspection of the data (Fig 2E) suggested possible modest decreases in phosphorylation in the mPFC, and a slight elevation of phosphorylation in the DH associated with clozapine treatment. The statistical analysis revealed a significant overall effect in the mPFC (maxT = 2.92, p = 0052), but not in the latPFC (maxT = 1.03, p = 0.71). A statistically significant planned comparisons indicated clozapine decreased Tyr816-TrkB phosphorylation in the mPFC (adjusted p = 0.020), whereas ketamine was associated with a non-significant trend for decreased phosphorylation in the same region (adjusted p = 0.072). The analysis did not detect any statistically significant effects in the hippocampal sub-regions (DH: maxT = 1.40, p = 0.49; VH: maxT = 1.03, p = 0.75). These result supported the idea that clozapine decreased the phosphorylation of Tyr816-TrkB in the mPFC.

For Ser2481-mTOR, we had collected phosphorylation data from the prefrontal cortex and hippocampus, using the same methods as described above. Data from the control and ketamine-alone groups of the mPFC and latPFC were reported in a previous compilation article, however [40]. Consequently, in Fig 2F we show only the hippocampal results. Visual inspection of the Ser2481-mTOR data suggested that both ketamine and clozapine may have increased its phosphorylation in the DH. This impression was confirmed by a statistically significant overall effect in the DH (maxT = 3.03, p = 0.0046), along with significant planned comparisons for both ketamine (adjusted p = 0.019) and clozapine (adjusted p = 0.006). No statistically significant effects related to the phosphorylation of Ser2481-mTOR were observed in the VH (maxT = 1.52, p = 0.39). Together these data indicated that both clozapine and ketamine increased Ser2481-mTOR phosphorylation in the DH, but not the VH.

For Thr202/Tyr204-ERK, visual inspection of the data (Fig 2G) suggested both ketamine and clozapine influenced its phosphorylation in the mPFC, while clozapine treatments appeared to be associated with increased phosphorylation in the hippocampus. The statistical analysis revealed a significant overall effect in the mPFC (maxT = 2.80, p = 0.012) and the planned comparisons confirmed that ketamine increased the phosphorylation (adjusted p = 0.0063), whereas clozapine treatment was associated with a non-significant trend in the same direction (adjusted p = 0.065). A significant overall effect was also detected in the latPFC (maxT = 2.58, p = 0.021), but the planned comparisons suggested only a non-significant trend for increased phosphorylation in the ketamine + clozapine group compared to the ketamine group (adjusted p = 0.067). There was also a significant overall effect in the DH (maxT = 2.72, p = 0.018), yet the planned comparison for the clozapine contrast was a non-significant trend (adjusted p = 0.066). No significant effects were detected in the VH (maxT = 1.09, p = 0.68). Overall, these results indicate that ketamine increased Thr202/Tyr204-ERK in the mPFC, but not in the other sub-regions. There was also some evidence suggesting clozapine may have done the same, but there was a high degree of variability in the clozapine-alone group.

While the above analysis of the phosphorylation data revealed several robust effects of ketamine and clozapine in prefrontal and hippocampal regions, the results did not provide clear evidence of a case where clozapine significantly reversed ketamine-induced phosphorylation. In the absence of such evidence, we performed an additional analysis of the data, which was directed at understanding the relative drug responsiveness of each phosphorylation residue in each brain region. That is, we were interested in estimating the *relative* impact of the ketamine and clozapine treatments on changes in phosphorylation. Effect size calculations provide a standardized estimate for quantifying differences between two groups [50]. Therefore, we calculated pairs of effect sizes for each residue in each region: (a) the effect size of the ketamine-alone groups compared to the respective saline controls, and (b) the effect sizes of the clozapine-alone groups compared to the respective saline controls. We then calculated a difference score of the absolute values of each effect size pair (difference score = |ketamine effect size|–|clozapine effect size|). The resulting difference scores where used as a metric for ranking the degree of relative responsiveness each residue exhibited in response to the two drugs, whereby positive scores suggested greater responsiveness to ketamine, and negative scores implied greater responsiveness to clozapine.

**Fig 3:**
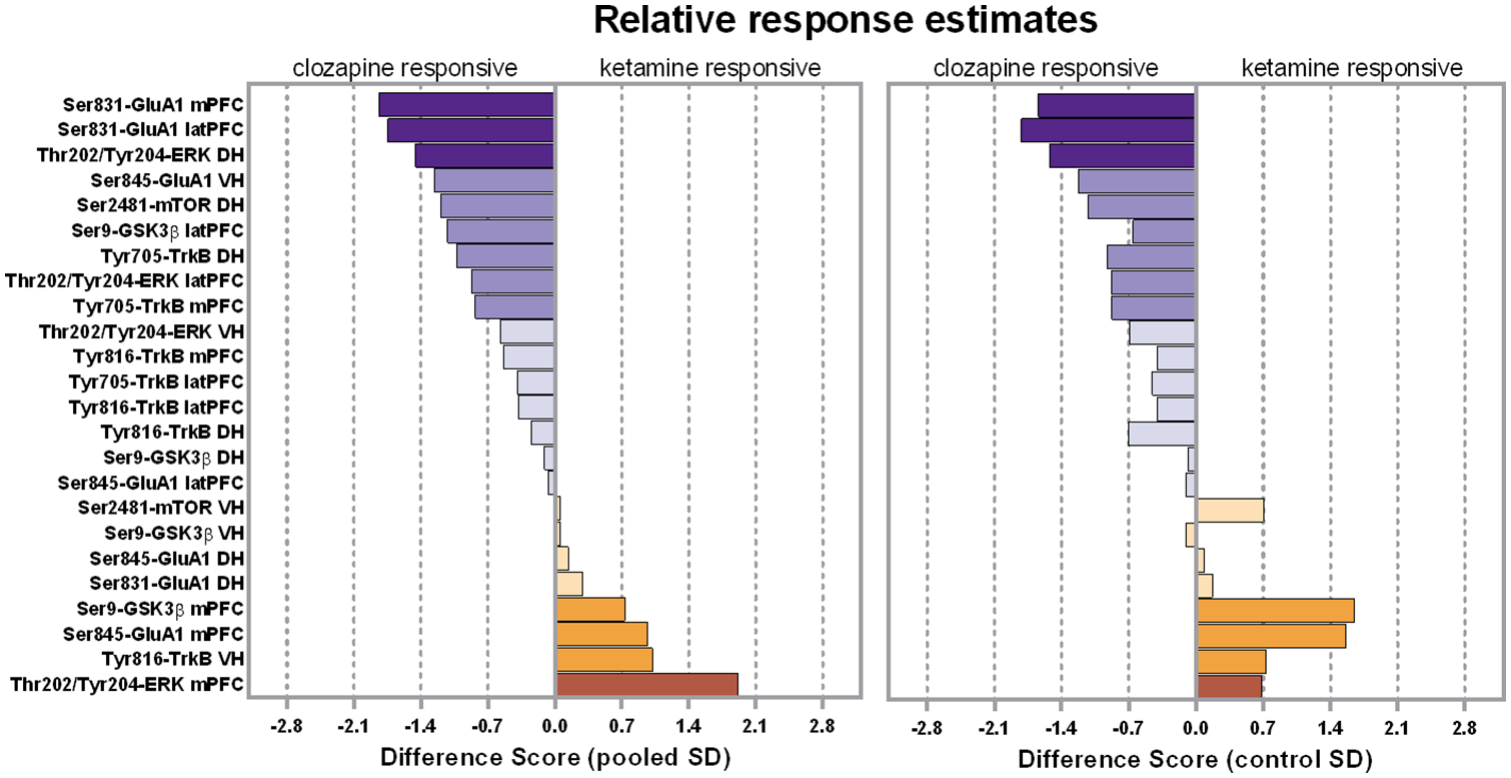
Relative drug response estimates. Difference between the ketamine and clozapine effect scores calculated for each phosphorylation residue in each sub-region. (Left panel) Difference scores when the effect size calculation used a pooled estimate of the standard deviation. (Right panel) Difference scores when the effect size calculation used the standard deviation of the control group. Negative values represent cases where the effect size of clozapine was greater than the effect size of ketamine. Positive values represent cases where the effect size of ketamine was greater than the effect size of clozapine.

The left panel of Fig 3 summarizes the difference scores that were calculated when a pooled estimate of the standard deviation was used. According to this estimate, the top four clozapine-responsive residues were Ser831-GluA1 in the mPFC, Ser831-GluA1 in the latPFC, Thr202/Tyr204-ERK in the DH and Ser845-GluA1 in the VH, while the top for ketamine-responsive residues were Thr202/Tyr204-ERK in the mPFC, Tyr816-TrkB in the VH, Ser845-GluA1 in the mPFC, and Ser9-GSK3β in the mPFC. Because in some situations it may be preferable to calculate effect sizes using only the standard deviation of the control group (instead of the pooled estimate), we also calculated a second series of effect sizes using the control groups standard deviation (Fig 3, right panel). Although magnitudes varied, for the majority of the groups the use of the alternative equation did not greatly alter the pattern of the relative effect size estimates. For example, the identities of the top four clozapine-responsive residues were the same as was estimated by the pooled equation, except magnitudes and ranks varied. For the ketamine-responsive residue, the four highest-ranked regions by the pooled estimate were ranked within the top five by the control estimate. All told, these estimates provide some indication for which phosphorylation residues exhibited the greatest relative responsiveness to the two drug treatments, with Ser831-GluA1 in the prefrontal cortex exhibiting the strongest clozapine responsivity relative to ketamine.

#### Total protein

**Fig 4:**
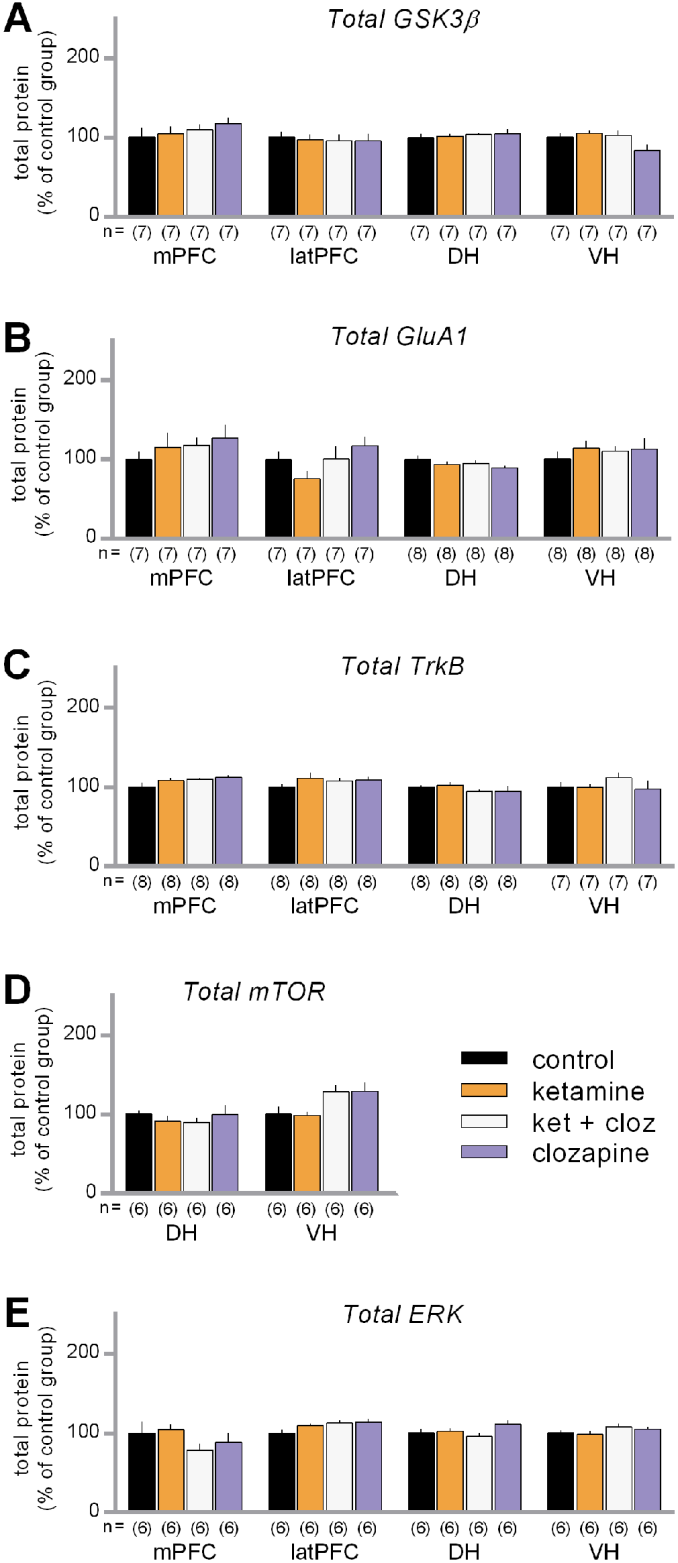
Influence of ketamine and clozapine on the total protein levels. Measurements were taken from tissue collected from the medial prefrontal cortex (mPFC), lateral prefrontal cortex (latPFC), dorsal hippocampus (DH) and ventral hippocampus (VH) 30 minutes after the drug treatments. (A) Average total levels of GSK3β. (B) Average total levels of GluA1. (C) Average total levels of TrkB. (D) Average total levels of mTOR. (E) Average total levels of ERK. Data are shown normalized with respect to the saline control group. The sample sizes of each group are shown underneath the x-axis. Error bars represent standard error of the means.

Visual inspection of the total protein data (Fig 4) suggested the possibility of some modest drug-induced changes in total protein levels. As with the phosphorylation data, the total protein data from each protein (split by sub-region) were entered into separate one-way permutation tests. These analyses detected a significant overall effect for GSK3β in the VH (maxT = 2.51, p = 0.037), and for ERK in the latPFC (maxT = 2.47, p = 0.038), as well as a non-significant trend for an overall effect for TrkB in the mPFC (maxT = 2.25, p = 0.081). None of the planned comparisons for these effects were significant, however (adjusted p-values: GSK3β_VH_ > 0.30; ERK_latPFC_ > 0.09). Also, all of the other protein/sub-region combinations had p-values > 0.13 with regard to the one-way overall effect. These results suggest that the drug treatments were not associated with reliable changes in total proteins levels for any of the proteins tested.

The data for these analyses are available in the available in the Supporting Information (S1 File).

## Discussion

In view of their respective effects on depressive and psychotic symptoms, the influence of ketamine and clozapine on the hippocampal-prefrontal circuit have each received much attention, but the mechanism underpinning clozapine’s counteraction of ketamine-induced effects is not well understood. The present results indicate that an acute low dose of ketamine decreased neural plasticity in the H-PFC pathway, whereas clozapine treatment normalized the ketamine-induced effect on the LTP. Furthermore, the two drugs exhibited several robust effects with regard to the phosphorylation of signaling molecules sampled from the prefrontal cortex and hippocampus. Ketamine significantly increased the phosphorylation of Ser9-GSK3β in the PFC (medial and lateral), Thr202/Tyr204-ERK in the mPFC, and Ser2481-mTOR in the DH. Clozapine increased the phosphorylation of Ser9-GSK3β and Ser831-GluA1 in prefrontal regions, Ser2481-mTOR in the DH, and Ser845-GluA1 in the VH, and it was also associated with a reduced phosphorylation of Tyr705-TrkB in the DH, and of Tyr816-TrkB in the mPFC.

Our electrophysiological results demonstrate that ketamine decreased the magnitude of *in vivo* LTP in the mPFC. On the one hand, ketamine is considered an “open-channel” blocker of the NMDA receptor [51] and H-PFC pathway LTP depends on NMDA receptor activation [16, 41], so this effect may be the direct consequence of ketamine’s allosteric influence on the NMDA receptor. On the other hand, ketamine also contributes to widespread changes in neurotransmitter release [35, 52–55] and brain activation, including alterations in functional connectivity of the hippocampal-prefrontal circuit [56, 57]. Notably, sub-anesthetic ketamine increases extracellular glutamate levels in the PFC [58], while NMDA antagonists are thought to disinhibit pyramidal cells by altering the drive of inhibitory interneurons in the region [59]. As such, the ketamine-induced depression of LTP we observed could stem from a form of metaplasticity, whereby elevated glutamatergic transmission preceding HFS may limit the capacity for subsequent LTP induction [60]. Ketamine blockade has also been observed to decrease spontaneous neurotransmission and CaMKII activity in the hippocampus [61], which could also underpin decreased potentiation in the H-PFC pathway. Previous work has also shown that a 20 mg/kg acute dose of clozapine augments LTP in the rat H-PFC pathway [62], whereas we observed that 0.3 mg/kg dose of clozapine did not appear to alter the LTP (S1 Fig). Clozapine has also been observed to increase spike amplitude in the rabbit perforant path at the 20 mg/kg dose, but not at 10 mg/kg [63]. Together, this pattern suggests that while clozapine may augment *in vivo* evoked potentials and plasticity in the hippocampal-prefrontal network, such effects appear to depend on higher dose treatments.

In regards to clozapine’s protection against the ketamine-induced depression of LTP, one possible explanation is that by augmenting the NMDA component of glutamatergic excitatory post-synaptic potentials [64] the low dose of clozapine counteracted the reduction of NMDA currents associated with ketamine’s modulation of NMDA receptor function. Alternatively, considering that ketamine decreases spontaneous activity, as well as CaMKII activation, in the hippocampus [61], there is a collection of evidence for the possibility of clozapine doing the opposite in the H-PFC network: Clozapine can act as a high-affinity antagonist for dopaminergic D4 receptors [65], which are expressed on GABAergic interneurons and in pyramidal output neurons throughout the rat frontal cortex [66]. Pharmacological blockade of D4 receptors can increase the excitability of PFC neurons [67], while D4 stimulation can lead to activity-dependent bi-directional regulation of AMPA transmission and trafficking in the PFC in a manner that depends on CaMKII [68]. Thus, clozapine could rescue LTP by boosting excitability in the H-PFC pathway, or by counteracting ketamine’s de-activation of CaMKII, which has been strongly implicated in synaptic plasticity [69]. It is also conceivable that clozapine operates through indirect routes involving alterations in AMPA receptor phosphorylation levels [70].

Our LTP results provide some evidence for the idea that H-PFC neuroplasticity has face validity for modeling those ketamine-clozapine interactions that have been observed with regard to the symptoms of schizophrenia [24, 25]. It is noteworthy that recent studies involving fMRI measurements show that there is a moderate degree of resting state coupling between the mPFC and the posterior hippocampus/subicular region in rats [71], which appears to be further increased by an acute dose of ketamine [56]. Importantly, however, in both healthy humans and rats [56, 57] the ketamine-induced increases in intra-PFC and hippocampal-prefrontal functional connectivity were nearly the opposite of the patterns observed in paranoid schizophrenic patients [72, 73]. Thus, considering that no one model typically recapitulates any human disease, ketamine’s influence on H-PFC plasticity may instead be most relevant for modeling the hyper-glutamatergic component of schizophrenia [56]. It could also be that H-PFC neuroplasticity is appropriate for modeling thought disorder and cognitive deficit symptoms of schizophrenia, but not paranoid symptoms.

The characterization of the intercellular signaling pathways that underpin the actions of antidepressant [48] and antipsychotic drugs [74] has been an important area of focus. One influential model has emphasized the role of the mTOR pathway in rapid anti-depressive actions of ketamine [47, 48]. According to this view, ketamine rapidly increases extracellular glutamate that contributes to depolarization and BDNF release, which in turn stimulates TrkB receptors. Activation of the TrkB receptors is then thought to stimulate multiple signaling cascades including the MEK-ERK and PI3K-ATK pathways, which then stimulate mTOR mediated signaling, leading to synaptogenesis and GluA1 insertion [47, 48]. Here we reported that ketamine increased Thr202/Tyr204-ERK and Ser9-GSK3β phosphorylation in the mPFC, while a previous report described the lack of an effect on mTOR phosphorylation [40]. The Thr202/Tyr204-ERK and Ser9-GSK3β phosphorylation observations are generally consistent with previous work [47, 75, 76], which supports the idea that ketamine activates ERK signaling and inhibits GSK3β activity. The cause of the seemingly discrepant mTOR results is not immediately clear, one possible explanation is related to dosing, since a subthreshold dose of ketamine was observed to increase ERK phosphorylation in the mPFC without affecting mTOR pathways [76]. We also observed that the relatively low dose of clozapine that we used was sufficient to induce robust increases in the phosphorylation of Ser831-GluA1 and Ser9-GSK3β throughout the prefrontal cortex. To our knowledge, such phosphorylation measurements of clozapine effects *in vivo* have previously only been reported for higher dose drug treatments, and so our results may better model clinical doses. Our Ser9-GSK3β results are similar to those observed for 5 mg/kg clozapine treatments [77], and they support the growing literature linking GSK signaling to clozapine and schizophrenia [78].

There are some notable parallels between our present and past results: both the antidepressant drug tianeptine and the antipsychotic drug clozapine can normalize the stress-induced disruption of LTP in the hippocampus and in the H-PFC pathway [10, 43, 49], and here we observed that clozapine can also normalize a ketamine-induced disruption of H-PFC LTP. We also demonstrated that clozapine increased the phosphorylation of Ser831-GluA1 in the mPFC, which was also the case for tianeptine [43], and both tianeptine and clozapine have been reported to augment post-synaptic NMDA currents with contribution from AMPA receptors [64, 79]. While these similarities do not prove how clozapine normalizes H-PFC LTP, they do provide an interesting target for future investigation. Moreover, these results lend credence to the idea that investigations of alterations in stress-sensitive H-PFC plasticity are useful for modeling the antidepressant and antipsychotic effects.

## Supporting information

S1 Fig Ketamine, but not clozapine, suppressed LTP in the H-PFC pathway. Data from animals treated with drug or saline injections 40 minutes prior to the induction of LTP in the mPFC via high-frequency stimulation (HFS) delivered to the ventral hippocampus/subicular region. (A) Timecourse data from animals treated with ip injections of saline or clozapine (0.3 mg/kg). (B) Mean PSP of the saline and clozapine groups averaged across the post-HFS interval. (C) Timecourse data from animals treated with iv injections of saline or ketamine (10 mg/kg). (D) Mean PSP of the saline and ketamine groups averaged across the post-HFS interval. Data were normalized with respect to the 30-minute baseline periods. Error bars represent standard error of the means. * indicates a statistically significant group difference based on a two-sided unpaired t-test.
(TIF)

S2 Fig Dose response of ketamine on H-PFC LTP. Summary data showing the average evoked post-synaptic potential (PSP) in groups of rats that were treated with saline or ketamine at three different doses (5, 10 and 25 mg/kg ip). (A) Mean post-HFS PSPs of rats that received a single dose 40 min prior to the induction of LTP. (B) Mean post-HFS PSPs of rats that received single daily treatments of sterile water (H_2_0) or ketamine for 14 days, and that were underwent testing on the 15th day. Data normalized as the percentage of baseline. Error bars represent standard error of the means. * indicates a significant group difference with respect to the vehicle control.
(TIF)

S3 Fig Dose response of phencyclidine on H-PFC LTP. Rats were given a single intravenous doses of saline (n = 6) or PCP (at 0.25, 0.50, or 1.00 mg/kg; n = 7 per group) 40 minutes prior to the induction of LTP. The HFS protocol produced a lasting increase in the amplitude of the PSP in all four groups, yet the middle and high dose groups appeared to have decreased levels of LTP compared to the saline control, especially during the first hour after induction. The post-HFS data were submitted to a repeated-measures ANOVA with a between-subject factor of Group and a within-subject variable of Block (four 30-min blocks). This analysis revealed statistically significant effects of Group (F_3,23_ = 3.12, p = 0.046) and of Block (F_3,69_ = 15.24, p < 0.00001), with a non-significant Group × Block interaction (F_9,69_ = 1.76, p = 0.092). Planned comparisons indicated that the high PCP dose (1.00 mg/kg) significantly depressed the induction of LTP. Also, although the 0.50 mg/kg PCP group appeared to be decreased during the first hour after HFS, this overall difference was not significant. These results indicate that a 1.0 mg/kg PCP dose depressed plasticity in the H-PFC pathway. (A) Timecourse data for each group showing average evoked PSP in 2 min bins. (B) Mean PSP of each group averaged across the post-HFS interval. For each group, data were normalized with respect to the 30-min baseline interval. Error bars represent standard error of the means. * indicates a statistically significant group difference compared to the saline control.
(TIF)

S4 Fig Representative images from western blots analysis. Immunoblots of the phosphorylated forms and total amounts of different target proteins in the four treatment conditions (control, ketamine, ketamine + clozapine and clozapine) and in different brain regions (mPFC, latPFC, DH and VH). Immunoblots illustrate, from top to bottom, P-Ser9-GSK3ß (46 kDa), GSK3ß (46 kDa), P-Ser831-GluA1 (100 kDa), P-Ser845-GluA1 (100 kDa), GluA1 (100 kDa), P-Tyr705-TrkB (145 kDa), P-Tyr816-TrkB (145 kDa), TrkB (90–145 kDa), P-Thr202/Tyr204-ERK (42 kDa), ERK (42–44 kDa), P-Ser2481-mTOR (289 kDa) and mTOR (289 kDa).
(TIF)

S1 File Data summary. (XLSX)
