# Supplementary figures and images for: Clozapine counteracts a ketamine-induced depression of hippocampal-prefrontal neuroplasticity and alters signaling pathway phosphorylation
Source: PLoS One. 2017 May 4;12(5):e0177036. doi: 10.1371/journal.pone.0177036 (PMC5417651; doi:10.1371/journal.pone.0177036)

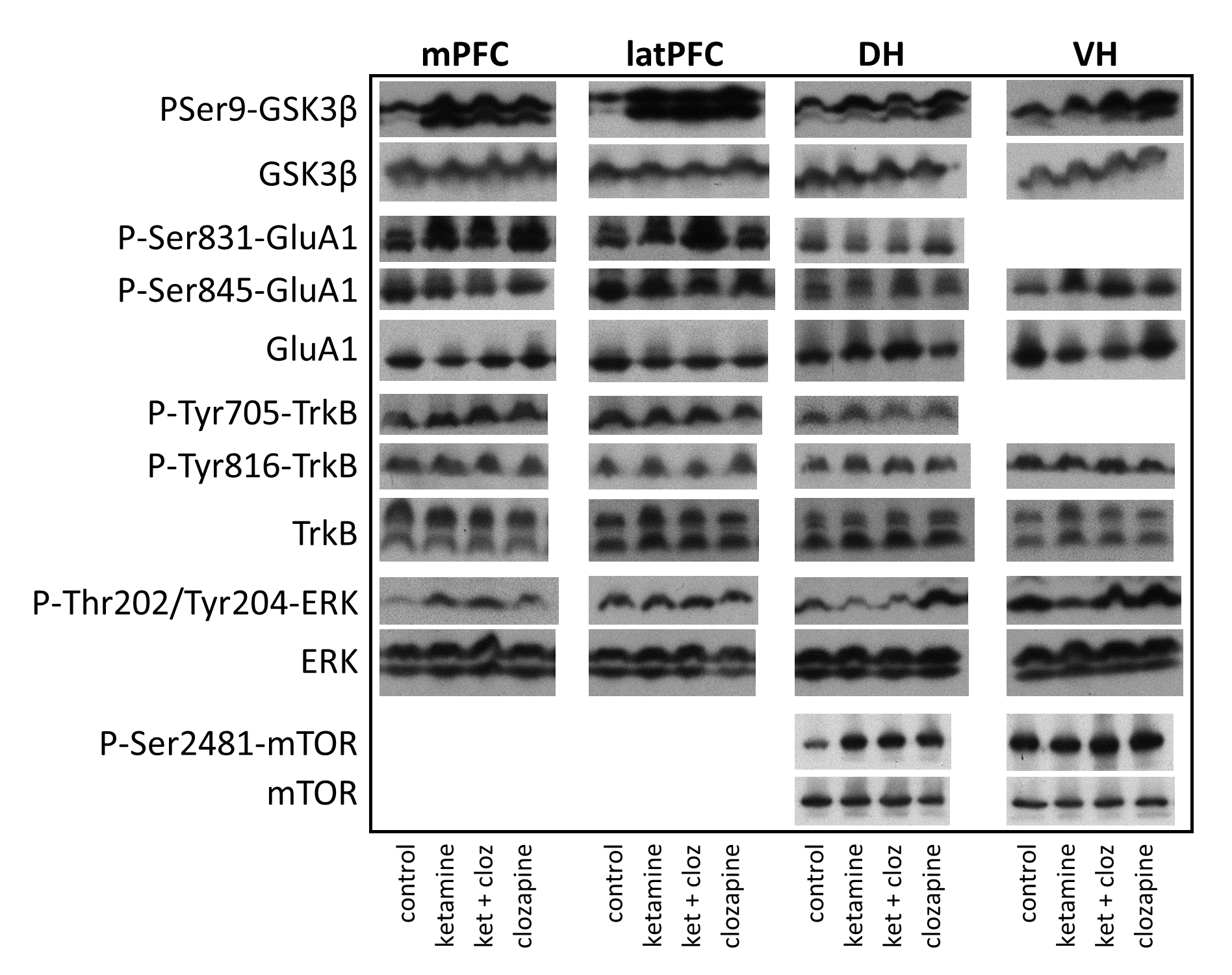

Supplement: S4 Fig — Immunoblots of the phosphorylated forms and total amounts of different target proteins in the four treatment conditions (control, ketamine, ketamine + clozapine and clozapine) and in different brain regions (mPFC, latPFC, DH and VH). Immunoblots illustrate, from top to bottom, P-Ser9-GSK3ß (46 kDa), GSK3ß (46 kDa), P-Ser831-GluA1 (100 kDa), P-Ser845-GluA1 (100 kDa), GluA1 (100 kDa), P-Tyr705-TrkB (145 kDa), P-Tyr816-TrkB (145 kDa), TrkB (90–145 kDa), P-Thr202/Tyr204-ERK (42 kDa), ERK (42–44 kDa), P-Ser2481-mTOR (289 kDa) and mTOR (289 kDa). (TIF) [file pone.0177036.s004.tif]
